# Disruption of genital ridge development causes aberrant primordial germ cell proliferation but does not affect their directional migration

**DOI:** 10.1186/1741-7007-11-22

**Published:** 2013-03-05

**Authors:** Su-Ren Chen, Qiao-Song Zheng, Yang Zhang, Fei Gao, Yi-Xun Liu

**Affiliations:** 1 State Key Laboratory of Reproductive Biology, Institute of Zoology, Chinese Academy of Sciences, 1 Beichen West Road, Chaoyang District, Beijing, 100101, China; 2 University of the Chinese Academy of Sciences, No.19A Yuquan Road, Shijingshan District, Beijing, 100049, China

**Keywords:** Primordial germ cells, Genital ridge, Migration, Proliferation

## Abstract

**Background:**

The directional migration and the following development of primordial germ cells (PGCs) during gonad formation are key steps for germline development. It has been proposed that the interaction between germ cells and genital ridge (GR) somatic cells plays essential roles in this process. However, the *in vivo *functional requirements of GR somatic cells in germ cell development are largely unknown.

**Results:**

*Wt1 *mutation (*Wt1*^R394W/R394W^) results in GR agenesis through mitotic arrest of coelomic epitheliums. In this study, we employed the GR-deficient mouse model, *Wt1*^R394W/R394W^, to investigate the roles of GR somatic cells in PGC migration and proliferation. We found that the number of PGCs was dramatically reduced in GR-deficient embryos at embryonic day (E) 11.5 and E12.5 due to decreased proliferation of PGCs, involving low levels of BMP signaling. In contrast, the germ cells in *Wt1*^R394W/R394W ^embryos were still mitotically active at E13.5, while all the germ cells in control embryos underwent mitotic arrest at this stage. Strikingly, the directional migration of PGCs was not affected by the absence of GR somatic cells. Most of the PGCs reached the mesenchyme under the coelomic epithelium at E10.5 and no ectopic PGCs were noted in GR-deficient embryos. However, the precise positioning of PGCs was disrupted.

**Conclusions:**

Our work provides *in vivo *evidence that the proliferation of germ cells is precisely regulated by GR somatic cells during different stages of gonad development. GR somatic cells are probably dispensable for the directional migration of PGCs, but they are required for precise positioning of PGCs at the final step of migration.

## Background

Primordial germ cells (PGCs) are the precursors of spermatozoa and oocytes, which are derived from a small number of epiblast cells under induction of bone morphogenetic protein (BMP) signaling and other unidentified signals from the extraembryonic ectoderm (ExE) and visceral endoderm (VE) at embryonic day (E) 6.5 in mice [1,2]. Following specification, PGCs must become motile and actively migrate across the embryo to reach the developing genital ridge (GR) and form the functional gonads in combination with surrounding somatic cells [3]. In mice, the PGCs move from the primitive streak to the endoderm at E7.5 [4]. Then, they migrate through the hindgut and mesentery and arrive at the GR at approximately E10.5 [5].

Previous studies have suggested that the directional migration of PGCs towards the GR is regulated by a combination of attractive and repulsive signals [6-8]. Stromal cell-derived factor 1 (*Sdf1*) is the most promising attractant signaling candidate, which is primarily expressed in the GR and surrounding mesenchyme; its receptor, *Cxcr4*, is expressed in PGCs [9-11]. The importance of this chemokine signaling for PGCs migration and colonization has been demonstrated by studying gene knockout mouse models [9,11]. *c-kit *is another gene that has been implicated in guiding mouse PGC migration. *c-kit *is expressed in PGCs and its ligand, *Steel factor*, is expressed by somatic cells along the migratory route [12]. Other than attractive signals, the adhesion molecule *E-cadherin *[13,14] and extracellular matrix molecule *Integrin β1 *[15] have been reported to be involved in the regulation of PGC migration and colonization in GR. However, the precise function of GR somatic cells in PGCs migration remains to be elucidated.

Once the PGCs reach the GR, they lose their motility and proliferate rapidly [16,17]. BMP signaling has been shown to control formation of the PGC niche and proliferation of PGCs within the early GR [18,19]. After sex determination, the germ cells in both male and female embryos cease proliferation. Male germ cells arrest at the G0 phase of mitosis, while female germ cells initiate meiosis and arrest at the diplotene stage of prophase I [20,21]. Whether the proliferation and differentiation of PGCs is a cell autonomous process [22-26] or is under the control of the surrounding gonadal somatic cells [27] remains an open question.

The Wilms' tumor (WT) suppressor gene, *Wt1*, encodes a nuclear zinc finger transcription factor that was originally identified as a tumor suppressor gene in patients with WT [28-30]. *Wt1 *has been implicated in the regulation of target genes related to proliferation and cell cycle progression [31,32]. *Wt1 *is expressed in the urogenital ridge coelomic epithelium and the underlying mesenchymal cells during embryo development [33]. It has been reported that *Wt1 *is essential for GR development and deletion of *Wt1 *results in gonadal agenesis due to the failure of GR development [33]. The alkaline phosphatase-positive PGCs are observed in the mesenchyme close to coelomic epithelium in *Wt1*^-/- ^embryo at E12, suggesting that aberrant GR development probably does not interfere with the germ cell migration [33]. However, whether the germ cell migrating process is normal and all the germ cells reach the aberrant GR; or the proliferation of germ cells has been affected in *Wt1*^-/- ^embryo remains unclear. *Wt1*^R394W ^mice carry the most common Denys-Drash syndrome (DDS) missense mutation [34]. *Wt1 +/*R394W mice display severe renal failure, and *Wt1*^R394W/R394W ^mice are embryonically lethal [34]. Agenesis of gonads and kidneys is also noted in *Wt1*^R394W/R394W ^mice due to aberrant GR development which is similar to *Wt1*-null mice.

In this study we found that the migrating process of PGCs was normal and no ectopic PGCs were observed in *Wt1*^R394W/R394W^. However, the number of PGCs in *Wt1*^R394W/R394W ^embryos was dramatically reduced compared to control embryos due to the mitotic arrest. In contrast, abnormal proliferation of PGCs was also observed in *Wt1*^R394W/R394W ^embryos at E13.5. These results suggest that the proliferation of germ cells during the early stage of gonad development is precisely regulated by GR somatic cells, and the GR somatic cells are probably dispensable for directional migration of PGCs.

## Results

### *Wt1 *mutation (*Wt1*^R394W/R394W^) results in GR agenesis through mitotic arrest of coelomic epitheliums

The GR arises as a thickening of the epithelium along the coelomic surface of the mesonephros [33,35]. A previous study suggests that *Wt1 *plays a critical role in GR development, as deletion of *Wt1 *results in aberrant GR development and absence of kidneys and gonads in mice [33]. The authors speculate that loss of *Wt1 *probably leads to apoptosis of coelomic epithelial cells, which in turn causes GR agenesis in *Wt1*^-/- ^mice [33]. However, the exact function of *Wt1 *in GR development is unclear.

**Figure 1:**
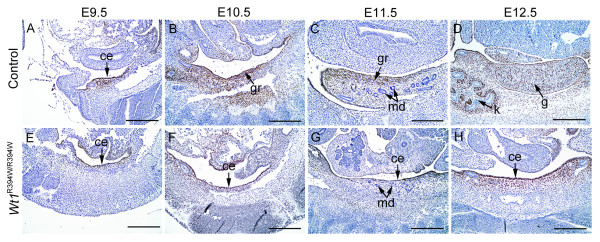
**Mutation of *Wt1 *resulted in GR agenesis**. WT1 (brown signal) was expressed in the coelomic epithelium and mesenchyme of the urogenital ridge from E9.5 (**A**, arrows) and continually expressed in somatic cells during gonad development (**B-D**). In control embryos, the GR arose as a thickening of the monolayer epithelium along the coelomic surface of the mesonephros from E9.5 and developed into gonads at E12.5 (A-D). The coelomic epithelium was present at E9.5 (**E**) in *Wt1*^R394W/R394W ^embryos, but further development was arrested (**F-G**, arrows), and no gonads were observed at E12.5 (F). ce, coelomic epithelium; gr, genital ridge; md, mesonephros duct; g, gonad; k, kidney. Scale bars: 50 μm.

**Figure 2:**
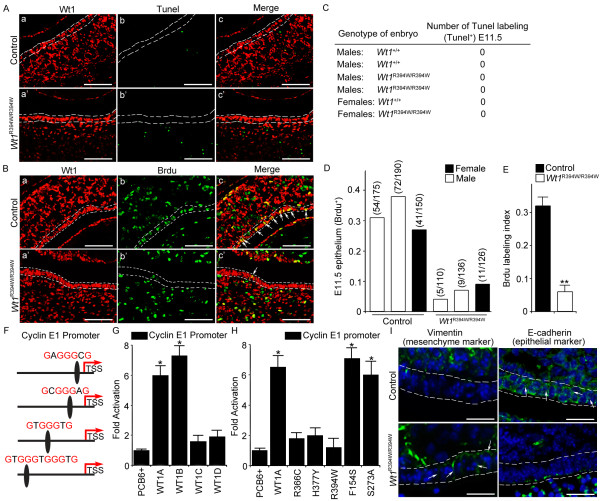
***Wt1 *is essential for epithelial cells characteristics maintenance and their normal proliferation**. TUNEL assay of coelomic epitheliums (white lines) was detected in *Wt1*^R394W/R394W ^embryo and control embryo (**A**). A table outlining the number of TUNEL^+ ^PGCs observed in control and *Wt1*^R394W/R394W ^E11.5 embryos (**C**). Proliferation of coelomic epitheliums (white lines) in *Wt1*^R394W/R394W ^embryos was examined, using BrdU incorporation assay (**B**). White arrows in B pointed to the BrdU^+ ^coelomic epitheliums. Scale bars: 30 μm. Numbers in parentheses show the actual number of BrdU^+ ^coelomic epitheliums over the total number of coelomic epitheliums counted for that embryo (**D**), and note that the BrdU labeling rate was significantly reduced in *Wt1*^R394W/R394W ^embryos (**E**). Four potential WT1 binding sites (GNGGGNG) in the cyclin E1 2.8-kb promoter were identified (**F**). TM4 cells were co-transfected with 0.2 μg cyclin E1-Luc (**G**), or 0.2 μg cyclin E1-Luc with different point mutants of the WT1A in modified PCB6+ vector (**H**), or the control PCB6+ vector. One group of mutants contained changes in the zinc finger region (R366C, H337Y and R394W), and the other group contained (F154S and S273A) changes outside that zinc finger region [37]. All of these assays were performed 36 hours after transfection. Fold activation was displayed by Firefly/Renilla ratio. Error bars represent SEM. *P <0.05 (by t-test). A significant elevation of mesenchymal markers vimentin (left) and a complete loss of the epithelial markers E-cadherin (right) and were detected by immunostaining assay (**I**). White arrows in I pointed to the signals. Scale bars: 30 μm.

In this study, *Wt1*^R394W ^point mutation mice strain [34] was used to study the interaction of GR somatic cells and germ cells in gonad development. It has been demonstrated that this mutation causes WT1 protein loss of function but does not affect its expression [34]; therefore, we can track the fate of coelomic epithelium, using the anti-WT1 antibody. We showed that WT1-positive coelomic epithelial cells were observed as early as E9.5 in both control (Figure 1A) and *Wt1*^R394W/R394W ^embryos (Figure 1E). However, further development of WT1-positive coelomic epithelial cells was arrested in *Wt1*^R394W/R394W ^embryos (Figure 1F-H). Surprisingly, the results of the terminal deoxynucleotidyl transferase-mediated deoxyuridine triphosphate nick endlabeling (TUNEL) assay showed that the number of apoptotic cells was not increased in WT1-positive coelomic epitheliums (white lines) of *Wt1*^R394W/R394W ^embryos compared to control embryos (Figure 2A, C), which was inconsistent with the previous study [33]. Further study revealed that the proliferation of coelomic epitheliums (white lines) in *Wt1*^R394W/R394W ^embryos was significantly reduced, using 5-Bromo-2-deoxyUridine (BrdU) incorporation assay (Figure 2B, D, E).

*Wt1 *has been implicated in the regulation of target genes related to cell cycle progression [31,32] and Cyclin E1 has been demonstrated to play a crucial role in the cell cycle by binding cycle-dependent kinase 2 (CDK2), which phosphorylates Rb, leading to transition from G1 into S phase [36]. To identify the underlying mechanism between *Wt1 *loss and mitotic arrest, we identified four potential WT1 binding sites within the cyclin E1 2.8-kb promoter (Figure 2F). The luciferase activity of TM4 cell transfected with WT1A or B (-KTS) was increased approximately 7-fold compared with the control. In contrast, no difference was observed between the control and WT1C or D (+KTS)-transfected TM4 cells (Figure 2G). To determine the effect of WT1(-KTS) in regulating *Cyclin E1 *promoter, we created two types of mutations in WT1A [37,38]. Mutants contained changes in the zinc finger region, such as R366C, H377Y, and R394W, failed to activate the *Cyclin E1 *promoter. In contrast, the mutants with mutations outside the zinc finger region, such as F154S and S273A, had transactivational potentials similar to that of wild-type WT1A (Figure 2H). Together, these results indicate that WT1 regulates the expression of *Cyclin E1*. Moreover, loss of function of *Wt1 *in GR somatic cells induced cell morphological changes characteristic of epithelial-mesenchymal transition, accompanied by a complete loss of the epithelial markers E-cadherin and a significant elevation of mesenchymal markers vimentin (Figure 2I). Taken together, these results indicate that the aberrant GR development in *Wt1*-mutant embryos is due to the mitotic arrest rather than apoptosis of coelomic epitheliums, and *Wt1 *is essential for *Cyclin E1 *regulation, epithelial cells characteristics maintenance and their normal proliferation.

### The directional migration of PGCs is not obviously affected in GR-deficient embryos

**Figure 3:**
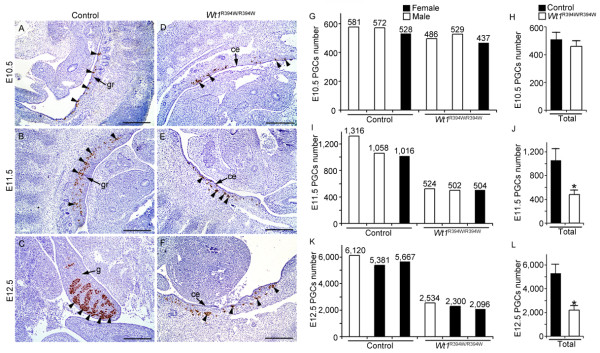
**The number of PGCs was reduced in GR-deficient embryos**. PGCs (black arrow-heads) were labeled with Stella (brown signal). At E10.5, Stella-positive PGCs were observed in the mesenchyme under the coelomic epithelium in both control (**A**) and *Wt1*^R394W/R394W ^(**D**) embryos, and no difference in PGC number was noted between control and *Wt1*^R394W/R394W ^embryos. In control embryos, Stella-positive PGCs colonized the developing gonads at E11.5 (**B**) and E12.5 (**C**), and the number of the PGCs increased rapidly. In contrast, in *Wt1*^R394W/R394W ^embryos, the PGCs were widely scattered in the mesenchyme under the coelomic epithelium at E11.5 (**E**) and E12.5 (**F**). Histogram summarizing selected PGCs number in microscopic cross sections of three independent embryos. Numbers in parentheses show the actual number of PGCs counted at E10.5 (**G**), E11.5 (**I**) and E12.5 (K). The average number of PGCs was significantly reduced in *Wt1*^R394W/R394W ^embryos compared with control embryos at these stages (**H, K, L**). * *P *<0.05. ce, coelomic epithelium; gr, genital ridge; g, gonad. Scale bars: 50 μm.

**Figure 4:**
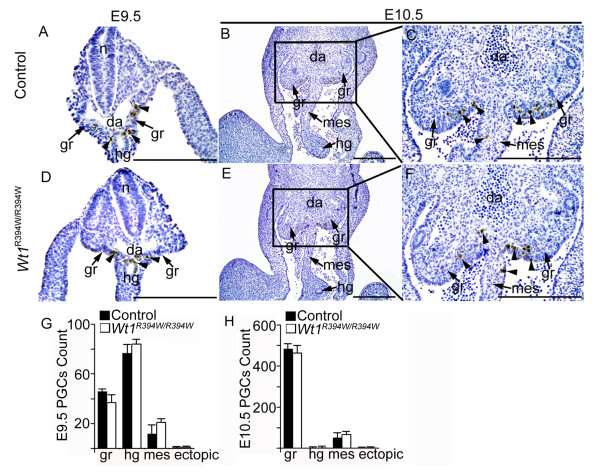
**PGC migration was normal in GR-deficient embryos**. Transverse sections of control (**A-C**) and *Wt1*^R394W/R394W ^embryos (**D-F**) were prepared and PGCs (black arrow-heads) were labeled with Stella (brown signal) (A-F). The PGC migration process was assessed by counting the number of PGCs at different locations along the migrating pathway at E9.5 (**G**) and E10.5 (**H**). At E9.5, most of the PGCs were observed in the hindgut and mesentery of both control (A) and *Wt1*^R394W/R394W ^(D) embryos. At E10.5, most of the PGCs had reached the GR, and very few PGCs remained in the mesentery (B, C, E, F). Histogram summarizing selected PGCs number in cross sections of three independent embryos and no difference was noted between control and *Wt1*^R394W/R394W ^embryos (**G, H**). n, neural tube; da, dorsal artery; gr, genital ridge; hg, hindgut; mes, mesentery. Scale bars: 50 μm.

Previous studies [8,35] have suggested that GR somatic cells participate in PGCs directional migration, although there is no direct *in vivo *evidence to support this hypothesis. To explore the roles of GR in PGC migration and later development, the PGCs migration process was attentively examined in control and *Wt1*^R394W/R394W ^embryos. As shown in Figure 3D, Stella-positive PGCs were observed in the mesenchyme near the coelomic epithelium in *Wt1*^R394W/R394W ^embryos, and the total number of PGCs in both male and female embryo was similar to that in control embryos at E10.5 (Figure 3G, H), using cross-section number counting of the whole embryo. To further detect the migration process of PGCs in *Wt1*^R394W/R394W ^embryos, cross sections of E9.5 and E10.5 embryos were prepared, and the numbers of PGCs at different locations along the migrating pathway were examined in Figure 4. At E9.5, most of the PGCs were observed in the hindgut and mesentery of both control (Figure 4A) and *Wt1*^R394W/R394W ^(Figure 4D) embryos. At E10.5, most of the PGCs reached the GR, and very few remained in the mesentery (Figure 4B, C, E, F). No difference of PGCs localization was noted between control and *Wt1*^R394W/R394W ^embryos by quantitative analysis (Figure 4G, H). In addition, no ectopic PGCs were detected in *Wt1*^R394W/R394W ^embryos. These results together indicate that functional GR is probably not essential for the directional migration of PGCs and that aberrant GR development does not affect PGC movement.

### The expression of Sdf1, Steel factor and Integrin-β1 was not changed in *Wt1*^R394W/R394W ^embryos

**Figure 5:**
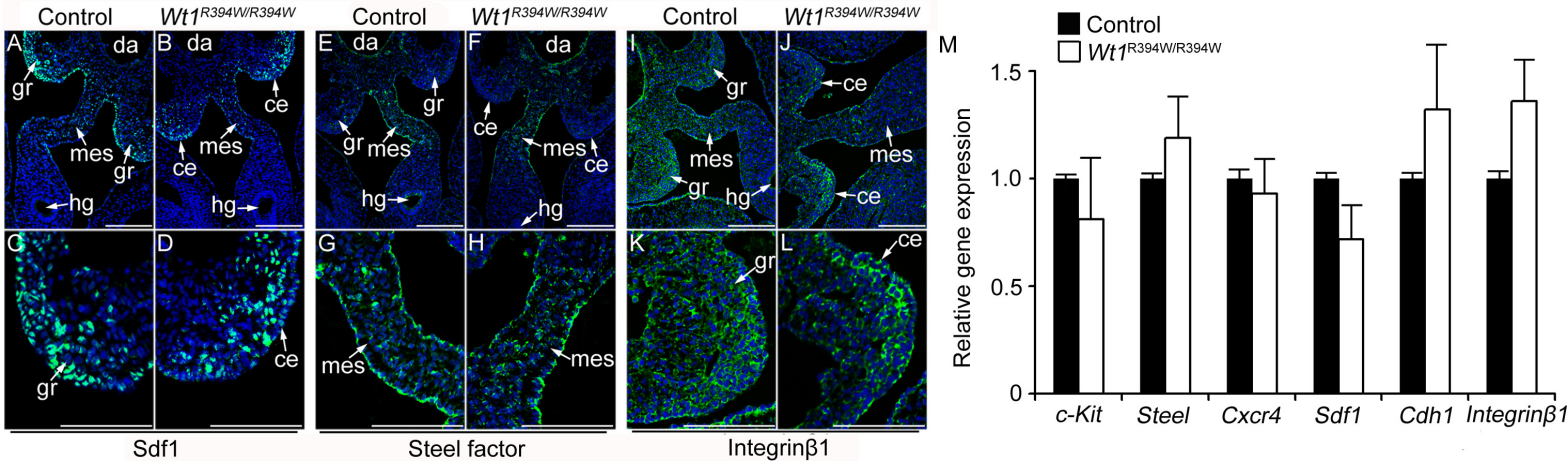
**Steel factor, Sdf1 and Integrin**β**1 expression were unchanged in GR-deficient embryos**. The expression patterns of *S*teel factor, Sdf1 and Integrinβ1 in E10.5 control and *Wt1*^R394W/R394W ^embryos were assessed by immunostaining. Sdf1 was expressed in the urogenital region of both control (**A**) and *Wt1*^R394W/R394W ^(**B**) embryos. High-magnification images showed that the expression of Sdf1 in the coelomic epithelium (white arrows) was not obviously changed in *Wt1*^R394W/R394W ^(**D**) compared with control embryos (**C**). Steel factor was detected in the mesentery of control (**E**) and *Wt1*^R394W/R394W ^(**F**) embryos. **G **and **H **are high-magnification images from E and F, respectively. Integrinβ1 was widely expressed in both control (**I**) and *Wt1*^R394W/R394W ^(**J**) embryos. **K **and **L **are high-magnification images of I and G, respectively. da, dorsal artery; gr, genital ridge; hg, hindgut; mes, mesentery. Scale bars: 50 μm. The mRNA level of *c-Kit, Steel, Cxcr4, Sdf1, Cdh1 *and *Integrinβ1 *was detected by real-time RT-PCR (**M**), and normalized against *Gapdh*. Each bar, mean ± SD of n = 3 experiments.

It has been demonstrated that excreted factors, such as Sdf1, Steel factor and Integrin-β1, involve in the directional migration of PGCs during gonad development [7,10,11,15,39]. To examine whether the expression of these factors is changed in GR-deficient embryos due to *Wt1 *mutation, immunofluorescence experiments at E10.5 were performed. As shown in Figure 5, the Sdf1 protein was expressed in the mesenchymal cells and coelomic epithelium in both control (Figure 5A) and *Wt1*^R394W/R394W ^(Figure 5B) embryos. High-magnification images showed that the expression of Sdf1 in the coelomic epithelium was not obviously changed in *Wt1*^R394W/R394W ^embryos (Figure 5D) compared with control embryos (Figure 5C). Steel factor was mainly expressed in the mesentery of both control (Figure 5E) and *Wt1*^R394W/R394W ^(Figure 5F) embryos. Integrin β1 was widely expressed in hindgut, mesentery and GR mesenchyme, and no difference was noted between control (Figure 5I, K) and *Wt1*^R394W/R394W ^(Figure 5J, L) embryos. Furthermore, we compared transcript levels of *c-Kit, Steel, Cxcr4, Sdf1, Cdh1 *and *Integrin-β1 *between control and *Wt1*^R394W/R394W ^embryos at E10.5. No differences were detected between samples for any of the genes using real-time RT-PCR (Figure 5M), indicating that the expression of factors which were implicated in regulating PGC migration was not changed in *Wt1*^R394W/R394W ^embryos.

### The proliferation of PGCs in the developing gonad is regulated by somatic cells

**Figure 6:**
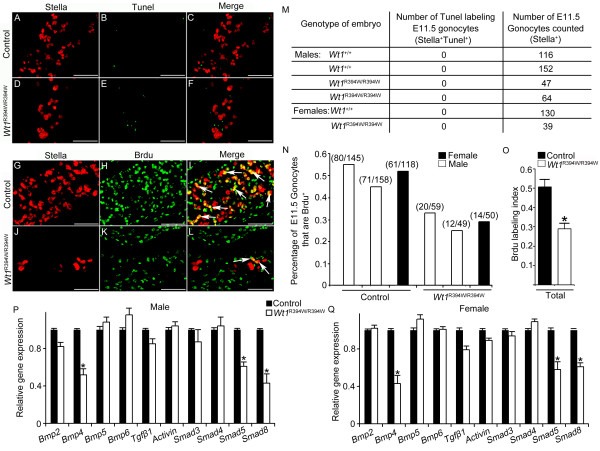
**PGC proliferation was decreased in GR-deficient embryos**. Cell apoptosis and proliferation were assessed by TUNEL (**A-F**, green) and BrdU incorporation (**G-L**, green) assays, respectively. The PGCs were labeled with Stella (red). Table outlining the number of TUNEL-positive PGCs observed in control and *Wt1*^R394W/R394W ^embryos (**M**). Note that no TUNEL-positive PGCs were observed in either control (C) or *Wt1*^R394W/R394W ^(F) embryos at E11.5. Percentage of total PGCs that are BrdU^+^. Numbers in parentheses show the actual number of BrdU^+ ^PGCs (Stella^+ ^BrdU^+ ^cells, white arrows) over the total number of PGCs counted for that embryo (Stella^+ ^cells) (**N**). Note that approximately 50% of the PGCs were labeled with BrdU (I, white arrows, **O**) in control embryos. In contrast, only approximately 28% of the PGCs in *Wt1*^R394W/R394W ^embryos were labeled with BrdU (L, white arrows, O). Scale bars: 30 μm. The mRNA level of BMP family members (*Bmp2, Bmp4, Bmp5 *and *Bmp6*), TGF-β family members (*Tgfβ1 *and *Activin*), and downstream genes of BMP signaling (*Smad3, Smad4, Smad5 *and *Smad8*) was detected by real-time RT-PCR in male (**P**) and female (**Q**). The mRNA level was normalized against *Gapdh*.* *P *<0.05. Each bar, mean ± SD of n = 3 experiments.

Upon reaching the GR, the PGCs proliferated rapidly and their number increased up to approximately 1,100 by E11.5 (Figure 3B, I) and 5,500 by E12.5 (Figure 3C, K). We found that the number of PGCs in *Wt1*^R394W/R394W ^embryos was significantly reduced compared to controls at E11.5 (Figure 3 E, I, J) and E12.5 (Figure 3F, K, L). To assess the reason for low PGCs number in *Wt1*^R394W/R394W ^embryos, TUNEL assay and BrdU incorporation experiments were conducted at E11.5. As shown in Figure 6, no TUNEL-positive PGCs were observed in control (Figure 6C, M) and *Wt1*^R394W/R394W ^(Figure 6F, M) embryos at E12.5. Numbers (parentheses) in Figure 6N showed the actual number of BrdU-positive PGCs over the total number of PGCs counted for that embryo. Note that approximately 50% of PGCs in control embryos were BrdU-positive (Figure 6I, N, O), while only approximately 28% of PGCs were BrdU-positive in *Wt1*^R394W/R394W ^(Figure 6L, N, O) embryos. These results indicate that the reduced number of PGCs in GR-deficient embryos is due to the mitotic arrest rather than apoptosis of PGCs. To further explore the molecular mechanism, we next assessed transcript levels for factors that may stimulate PGCs proliferation within E11.5 gonad. Potential factors include the BMP family members and TGF-β family members. The BMP family has been associated with PGCs proliferation within the early GR [18,19,40] and two members of the TGF-β family, TGFβ1 and activin, limited murine PGCs proliferation [41,42]. *Bmp4 *and *Smads *were validated as WT1 targets by ChIP-PCR in kidney [43], suggesting that *Bmp4 *might most likely be transcriptional targets of WT1. Using real-time RT-PCR, we detected that the mRNA levels of *Bmp4 *and downstream genes of BMP signaling, including *Smad5 *and *Smad8*, were significantly reduced in both male and female *Wt1*^R394W/R394W ^embryos (Figure 6P, Q). So, we conclude that the low level of BMP signaling within the GR is one of the reasons accounted for mitotic arrest and low PGCs number in *Wt1*-mutant mice.

**Figure 7:**
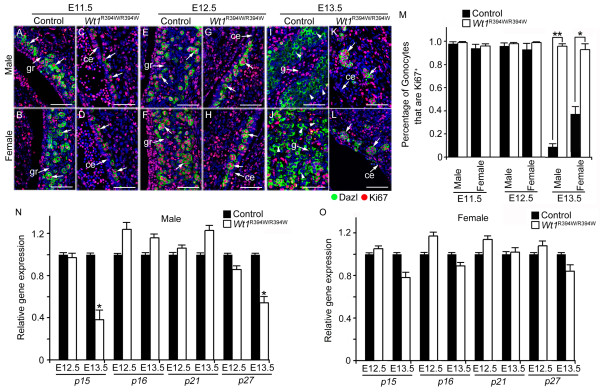
**Abnormal germ cell proliferation was noted in GR-deficient embryos after sex determination**. PGCs were labeled with Dazl (green), and proliferating cells were labeled with Ki67 (red). Most of the germ cells were Ki67-positive at E10.5 (**A**, **B**) and E11.5 (**E**, **F**), but the Ki67 signal was absent in most germ cells in control embryos after sex determination at E13.5 (**I**, **J**, white arrow-heads). In *Wt1*^R394W/R394W ^embryos, germ cells were labeled with Ki67 at E10.5 (**C**, **D**) and E11.5 (**G**, **H**), similar to control embryos. However, the germ cells in *Wt1*^R394W/R394W ^embryos remained Ki67-positive (**K**, **L**, white arrows) at E13.5. ce, coelomic epithelium; gr, genital ridge; g, gonad. Scale bars: 30 μm. Histogram summarizing selected percentage of gonocytes that were Ki67^+ ^and note that the percentage of Ki67^+ ^gonocytes were significantly increased in *Wt1*^R394W/R394W ^embryos at E13.5, compared with control embryos at the same stage (**M**). The mRNA level of CDKI (*p15*^INK4b^, *p16*^Ink4A^, *P21*^WAF1/Cip1 ^and *p27*^Kip1^) that inhibited the G1/S transition was estimated by real-time RT-PCR at E12.5 and E13.5 and normalized against PGC-specific marker *Stella *(**N**, **O**). * *P *<0.05, * * *P *<0.01. Each bar, mean ± SD of n = 3 experiments.

After a rapid increase in number, the PGCs cease proliferating after sex determination [20,21]. As shown in Figure 7, most of the Dazl-positive (green) germ cells were labeled with Ki67 at E11.5 (Figure 7A, B) and E12.5 (Figure 7E, F) in control embryos. However, very few Ki67-positive male germ cells were noted at E13.5 (Figure 7I, M), indicating that male germ cells in control embryos are in mitotic arrest at this stage. The percentage of Ki67-positive female germ cells was approximately 35%, while approximately 96% of germ cells in *Wt1*^R394W/R394W ^embryos remained Ki67-positive at E13.5 (Figure 7K, L, M; white arrows). Because a high percentage of germ cells in *Wt1*^R394W/R394W ^embryos does not leave active cell cycle process, it is likely that the cyclin-dependent kinase inhibitor (CDKI) that inhibited the G1/S transition was relatively down-regulated. We found that expression of *p15*^INK4b ^and *p27*^Kip1^, as estimated by real-time RT-PCR, was down-regulated in *Wt1*^R394W/R394W ^germ cells, compared with male germ cells in control (Figure 7N). In contrast, there were no significant differences in transcripts of *p15*^INK4b ^and *p27*^Kip1 ^between female germ cells in control and *Wt1*^R394W/R394W ^germ cells (Figure 7O). These results together suggest that the proliferation of germ cells during gonad development is precisely regulated by somatic cells and that the aberrant GR development results in abnormal germ cell proliferation.

### The PGC identity and PGC-PGC interactions are not changed in *Wt1*^R394W/R394W ^embryos

**Figure 8:**
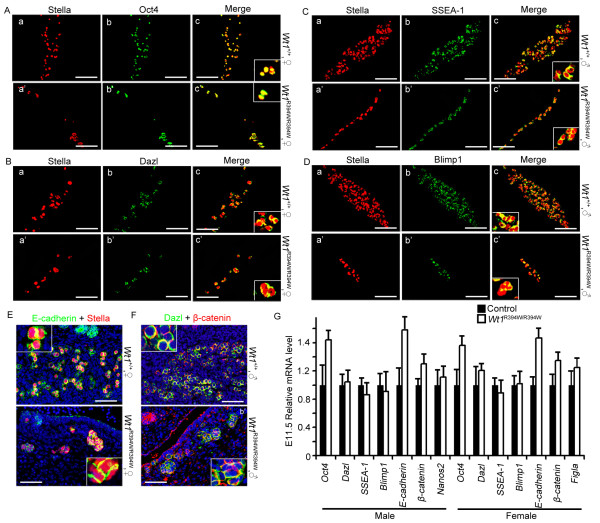
**The PGCs identity was not changed in *Wt1*^R394W/R394W ^embryos**. PGCs identity was examined by double staining of Stella (red) with PGCs characteristic markers, including Oct4 (**A**), Dazl (**B**), SSEA-1 (**C**), and Blimp1 (**D**). In *Wt1*^R394W/R394W ^embryos, PGCs still expressed Stella, SSEA-1, Blimp1, Dazl and pluripotent marker Oct4. Scale bars: 50 μm. PGCs-PGCs interactions, such as E-cadherin and β-catenin were maintained in *Wt1*^R394W/R394W ^embryos (**E**, **F**). Scale bars: 50 μm. The mRNA level of PGCs characteristic markers (*Oct4, Dazl, SSEA-1, Blimp1, Nanos2, Figla*), and PGCs-PGCs interaction markers (*E-cadherin *and *β-catenin*) were detected by real-time RT-PCR, and normalized against PGC-specific marker Stella (**G**). Each bar, mean ± SD of n = 3 experiments.

It has been found that PGCs reside in extragonadal tissues, such as the adrenal and mesonephric tissues in some case [44]. The identity of these ectopic PGCs may partially change [16,45,46]. In our study, the PGCs settled under the epithelium of mesonephros. We questioned whether the PGC identity changed without the support of GR somatic cells. To test, the PGC identity was detected in E11.5 *Wt1*^R394W/R394W ^embryos. We found that PGCs in both male and female GR-absent embryos still expressed PGC characteristic markers, including Stella, SSEA-1, Blimp1, Dazl and the pluripotent marker Oct4 (Figure 8A-D, 8G). In addition, PGC-PGC interactions, such as E-cadherin and β-catenin were maintained in E11.5 *Wt1*^R394W/R394W ^embryos (Figure 8E, F). These results suggest that the PGC identity and important characteristics do not alter in *Wt1*^R394W/R394W ^embryos. The mRNA level of PGC characteristic markers (*Oct4, Dazl, SSEA-1, Blimp1, Nanos2, Figla*), and PGC-PGC interaction markers (*E-cadherin *and *β-catenin*) were not changed between control and *Wt1*^R394W/R394W ^embryos, detected by real-time RT-PCR, and normalized against the PGC-specific marker Stella.

## Discussion

The PGCs are formed distantly from their final destination, thus, they must migrate for a long distance to reach the GR. The precise mechanism that regulates the directional migration of PGCs towards the GR remains an open question [47]. The widely accepted hypothesis suggests that the PGCs are attracted by the factors that are emitted from the destination or by somatic cells along their migratory route. *In vitro *studies have demonstrated that the GR tissue from E10.5 embryos attracts PGCs migration [35]. Several genes have been implicated in the process of PGC migration, such as *Sdf-1, Steel factor, Integrinβ1 *[8,15] and *E-cadherin *[14], and the inactivation of these genes results in aberrant PGC migration. In this study, a GR-deficient mouse model, *Wt1*^R394W/R394W^, was used to study the roles of the GR in germ cell migration and development. We found that the migration of PGCs in GR-deficient embryos was normal, which was consistent with a previous study [33]. Stella-positive PGCs reached the mesenchyme under the coelomic epithelium at E10.5, and no ectopic PGCs were observed in GR-deficient embryos. However, the number of PGCs was dramatically reduced in GR-deficient at E11.5 and E12.5. Further study revealed that the decrease of PGC number was due to the reduced proliferation but not cell apoptosis.

Sdf1, Steel factor and Integrinβ1 have been reported to play important roles in regulating PGC migration [8,15]. In this study, we found that the expression of these proteins was not restricted to GR somatic cells and the protein level was also not changed in *Wt1*^R394W/R394W ^embryos compared to control embryos. These results suggest that the signals that regulate PGC migration probably not only come from GR somatic cells, but the factors from hindgut, mesentery and the mesenchymal cells around GR also play important roles in this process. Given that PGCs begin to migrate at approximately E7.5, while the GR is not visible until approximately E9.5 in mice, the early stage of PGC migration is most likely regulated by somatic cells along the migrating pathway, not by signals from the GR. In this study, we found that the GR development was blocked in *Wt1*^R394W/R394W ^embryos due to *Wt1 *mutation. However, the coelomic epithelium was still maintained from E9.5 to E12.5. We could not exclude the possibility that the developmentally arrested coelomic epithelial cells still can secret some unknown factors which attract the PGC migration, and other functional GR is not essential for germ cell migration. We also found that most of the PGCs in control and *Wt1*^R394W/R394W ^embryos arrived at the mesenchyme near the coelomic epithelium at E10.5. However, at E11.5 and E12.5, all the germ cells in control embryos were colonized into the developing GR (Figure 3B, C), whereas, the germ cells in GR-deficient embryos were scattered widely under the coelomic epithelium instead (Figure 3E, F). These results suggest that GR somatic cells are important for the precise positioning of PGCs at the final step of migration.

During and after colonization into the GR, PGCs proliferate rapidly, and their number is increased dramatically from approximately 500 to 25,000 by E13.5 [48,49]. After sex determination, most germ cells enter mitotic arrest [20,21]. Whether the proliferation of germ cells is a cell autonomous process or regulated by GR somatic cells is unclear. In this study, we found that the proliferation of PGCs was dramatically reduced in *Wt1*^R394W/R394W ^embryos at E11.5 and E12.5 compared to control embryos, whereas, the germ cells in *Wt1*^R394W/R394W ^embryos were still mitotically active at E13.5 when all the germ cells stop proliferation in control embryos, indicating that the proliferation of germ cells is precisely regulated by GR somatic cells during the early stage of gonad development, and disruption of GR development results in aberrant germ cell proliferation. Our results were consistent with previous *in vitro *studies, which showed that soluble factors released by GR regulated the proliferation of PGCs using the medium conditioned by different embryonic tissues [35].

## Conclusions

The results of our study provide *in vivo *evidence that the proliferation of germ cells is tightly regulated by GR somatic cells during the early stage of gonad development. The directional migration of PGCs is not affected, but the precise positioning of PGCs at the final step of migration is disrupted in GR-deficient embryos, suggesting that the functional GR is not essential for PGC migration. However, the detailed molecular mechanisms underlying this regulation await further investigation.

## Methods

### Mice

All animal work was approved by the committee on animal care at the Institute of Zoology of the Chinese Academy of Sciences. All mice were maintained in a C57BL/6; 129/SvEv mixed background. *Wt1*^R394W/R394W ^mice were obtained by crossing male and female *Wt1*^+/R394W ^mice [34]. DNA isolated from adult tails and fetal tissues was used for genotyping. Genotyping was performed by PCR as described previously [34]. The sex of the embryo was detected by PCR using *Sry *primer [50].

### Immunohistochemistry and immunofluorescence

Embryos were collected at E9.5 to E13.5. Embryos were dissected in PBS and fixed in 4% paraformaldehyde for up to 24 hours, stored in 70% ethanol, and embedded in paraffin. Tissue sections (5 μm thick) were cut and mounted on glass slides. Sections were deparaffinized and rehydrated, followed by antigen retrieval in 10 mM sodium citrate buffer. After blocking with 5% BSA for one hour, the sections were incubated with primary antibody at 4°C overnight. After washing with PBS, secondary antibody was applied for one hour, followed by washing in PBS. Staining was visualized using a DAB substrate kit (Zhong Shan Technology, Beijing, China). For immunofluorescence, the sections were blocked in blocking buffer (goat serum, 0.3% Triton X-100 in PBS) at room temperature for one hour and then incubated with primary antibodies overnight at 4°C. Sections were washed in 0.3% Triton X-100 in PBS and incubated with FITC or TRITC-conjugated secondary antibodies (Jackson ImmunoResearch, West Grove, PA, USA) for one hour. The sections were washed in 0.3% Triton X-100 in PBS and counterstained with 4',6-diamidino-2-phenylindole (DAPI) (Sigma, St. Louis, MO, USA) to identify the nuclei.

The following antibodies were used in this study: rabbit anti-WT1 (1/400, Epitomics, Burlingame, CA, USA, 2797-1), rabbit anti-Stella (1/200, Santa Cruz Biotechnology, Santa Cruz, CA, USA, sc-67249), rabbit anti-Ki67 (1/500, Abcam, Cambridge, MA, USA, ab15580), mouse anti-Dazl (1/400, AbD Serotec, Raleigh, NC, USA, MCA2336), goat anti-Steel (1/50, Santa Cruz Biotechnology, sc-1303), rabbit anti-Sdf1 (1/50, Santa Cruz Biotechnology, sc-28876), rabbit anti-Integrinβ1 (1/500, Chemicon, Billerica, MA, USA, AB1952), rabbit anti-E-cadherin (1/200, Abcam, ab15148), and rabbit anti-Vimentin (1/200, Cell Signaling Technology, Danvers, MA, USA, #3932).

### Count PGC number

A serial section of the whole embryo was prepared and stained by PGC-specific marker Stella. The Stella^+ ^cells were counted at each section and added to the total PGC number of that embryo.

### TUNEL assay

TUNEL assays were conducted with the *In Situ *Cell Death Detection Kit, Fluorescein (Promega BioSciences, San Luis Obispo, CA, USA), as recommended. Images were acquired with a Nikon DMR Epifluorescence Microscope (Nikon Instruments Inc, Melville, NY, USA), and images were captured by a Hamamatsu CCD camera (Hamamatsu Photonics, Iwata, Shizuoka, Japan).

### BrdU incorporation assay

BrdU labeling and detection were conducted as previously described [51]. Briefly, pregnant females were injected with 50 mg/kg of BrdU (Sigma) (intraperitoneally, i.p.) one hour prior to embryo collection. Embryos were dissected and fixed in 4% PFA. A purified mouse anti-BrdU monoclonal antibody (BD Bioscience, San Jose, CA, USA) was used for BrdU detection.

### Real-time RT-PCR

The genital ridge were lyzed with Trizol reagent (Invitrogen, Carlsbad, CA, USA) and total RNA was extracted according to the manufacturer's instructions. Measurement of RNA integrity and cDNA synthesis of cDNA were performed as previously described [52]. Each sample was measured in duplicate in at least three independent experiments. Sample CT values were normalized to the corresponding *Gapdh *or *Stella *CT values, and relative expression levels were calculated using the ΔΔCT method [25]. Primer pairs were listed in Additional file 1, Table S1.

### Plasmid construction and cell transfection

Mouse *Wt1 *cDNA was amplified by PCR using testis cDNA and subcloned into the PCB6+ vector, as described [38]. The 2.8-kb cyclin E1 promoter fragment was amplified by PCR from mouse genomic DNA and subcloned into the pGL3-basic luciferase reporter vector (Promega). Point mutation of WT1A was performed by site-directed mutagenic PCR, using the PCB6+WT1A construct as the template [38]. TM4 cells were grown in F-12/DMEM supplemented with 10% fetal calf serum and cells were co-transfected with expression and reporter plasmids and the Renilla luciferase reporter plasmid (pRL-TK) (Promega) as indicated in the figure legends. The cells were harvested for the luciferase assay 36 hours after transfection.

### Statistical analysis

Experiments were repeated at least three times. The data were evaluated for significant differences using a Student's *t*-test. A *P*-value <0.05 (*) was considered significant. A *P*-value <0.01(**) was considered very significant. Bar graphs were plotted in MS Excel (Microsoft Corporation, Redmond, WA, USA).

## Abbreviations

BMP, Bone morphogenetic protein; BrdU, 5-Bromo-2-deoxyUridine; BSA, Bovine serum albumin; CDK2, Cycle-dependent kinase 2; CDKI, Cyclin-dependent kinase inhibitor; DAB, Diaminobenzidine; DAPI, 4',6-diamidino-2-phenylindole; DDS, Denys-Drash syndrome; DMEM, Dulbecco's Modified Eagle Medium; E, Embryonic day; ExE, Extraembryonic ectoderm; FITC, Fluorescein isothiocyanate; GR, Genital ridge; PBS, Phosphate-buffered saline; PCR, Polymerase chain reaction; PGCs, Primordial germ cells; *Sdf1*, Stromal cell-derived factor 1; TUNEL, Terminal deoxynucleotidyl transferase-mediated deoxyuridine triphosphate nick endlabeling; VE, Visceral endoderm; WT1, Wilms' tumor 1

## Competing interests

The authors declare that they have no competing interests.

## Authors' contributions

FG and YL conceived and designed the study. SC performed all the experiments, analyzed data and wrote the manuscript. QZ and YZ helped to analyze data. All authors have read and approved the manuscript for publication.

## Supplementary Material

Additional file 1 **Table S1: DNA primers used in this study**. Primers used in this study for Real-time RT-PCR. Click here for file

## References

[B1] Saga Y Mouse germ cell development during embryogenesis Curr Opin Genet Dev 2008 18 337 341 10.1016/j.gde.2008.06.003 18625315

[B2] Starz-Gaiano M Lehmann R Moving towards the next generation Mech Dev 2001 105 5 18 10.1016/S0925-4773(01)00392-6 11429277

[B3] Santos AC Lehmann R Germ cell specification and migration in *Drosophila *and beyond Curr Biol 2004 14 R578 589 10.1016/j.cub.2004.07.018 15268881

[B4] Anderson R Copeland TK Scholer H Heasman J Wylie C The onset of germ cell migration in the mouse embryo Mech Dev 2000 91 61 68 10.1016/S0925-4773(99)00271-3 10704831

[B5] Molyneaux KA Stallock J Schaible K Wylie C Time-lapse analysis of living mouse germ cell migration Dev Biol 2001 240 488 498 10.1006/dbio.2001.0436 11784078

[B6] Molyneaux K Wylie C Primordial germ cell migration Int J Dev Biol 2004 48 537 544 10.1387/ijdb.041833km 15349828

[B7] Raz E Guidance of primordial germ cell migration Curr Opin Cell Biol 2004 16 169 173 10.1016/j.ceb.2004.01.004 15196560

[B8] Richardson BE Lehmann R Mechanisms guiding primordial germ cell migration: strategies from different organisms Nat Rev Mol Cell Biol 2010 11 37 49 10.1038/nrm2815 20027186 PMC4521894

[B9] Ara T Nakamura Y Egawa T Sugiyama T Abe K Kishimoto T Matsui Y Nagasawa T Impaired colonization of the gonads by primordial germ cells in mice lacking a chemokine, stromal cell-derived factor-1 (SDF-1) Proc Natl Acad Sci USA 2003 100 5319 5323 10.1073/pnas.0730719100 12684531 PMC154343

[B10] Bacon K Baggiolini M Broxmeyer H Horuk R Lindley I Mantovani A Matsushima K Murphy P Nomiyama N Oppenheim J Rot A Schall T Tsang M Thorpe R Van Damme J Wadhwa M Yoshie O Zlotnik A Zoon K Chemokine/chemokine receptor nomenclature Cytokine 2003 21 48 4912668160

[B11] Molyneaux KA Zinszner H Kunwar PS Schaible K Stebler J Sunshine MJ O'Brien W Raz E Littman D Wylie C Lehmann R The chemokine SDF1/CXCL12 and its receptor CXCR4 regulate mouse germ cell migration and survival Development 2003 130 4279 4286 10.1242/dev.00640 12900445

[B12] Matsui Y Zsebo KM Hogan BL Embryonic expression of a haematopoietic growth factor encoded by the Sl locus and the ligand for c-kit Nature 1990 347 667 669 10.1038/347667a0 1699134

[B13] Bendel-Stenzel MR Gomperts M Anderson R Heasman J Wylie C The role of cadherins during primordial germ cell migration and early gonad formation in the mouse Mech Dev 2000 91 143 152 10.1016/S0925-4773(99)00287-7 10704839

[B14] Di Carlo A De Felici M A role for E-cadherin in mouse primordial germ cell development Dev Biol 2000 226 209 219 10.1006/dbio.2000.9861 11023681

[B15] Anderson R Fassler R Georges-Labouesse E Hynes RO Bader BL Kreidberg JA Schaible K Heasman J Wylie C Mouse primordial germ cells lacking beta 1 integrins enter the germline but fail to migrate normally to the gonads Development 1999 126 1655 1664 10079228 10.1242/dev.126.8.1655

[B16] Donovan PJ Stott D Cairns LA Heasman J Wylie CC Migratory and postmigratory mouse primordial germ cells behave differently in culture Cell 1986 44 831 838 10.1016/0092-8674(86)90005-X 3955652

[B17] Enders GC May JJ Developmentally regulated expression of a mouse germ cell nuclear antigen examined from embryonic day 11 to adult in male and female mice Dev Biol 1994 163 331 340 10.1006/dbio.1994.1152 8200475

[B18] Dudley B Palumbo C Nalepka J Molyneaux K BMP signaling controls formation of a primordial germ cell niche within the early genital ridges Dev Biol 2010 343 84 93 10.1016/j.ydbio.2010.04.011 20417197 PMC2885459

[B19] Farini D Scaldaferri ML Iona S La Sala G De Felici M Growth factors sustain primordial germ cell survival, proliferation and entering into meiosis in the absence of somatic cells Dev Biol 2005 285 49 56 10.1016/j.ydbio.2005.06.036 16139834

[B20] Menke DB Koubova J Page DC Sexual differentiation of germ cells in XX mouse gonads occurs in an anterior-to-posterior wave Dev Biol 2003 262 303 312 10.1016/S0012-1606(03)00391-9 14550793

[B21] Suzuki A Saga Y Nanos2 suppresses meiosis and promotes male germ cell differentiation Genes Dev 2008 22 430 435 10.1101/gad.1612708 18281459 PMC2238665

[B22] Agoulnik AI Lu BS Zhu QC Truong C Ty MT Arango N Chada KK Bishop CE A novel gene, Pog, is necessary for primordial germ cell proliferation in the mouse and underlies the germ cell deficient mutation, gcd Hum Mol Genet 2002 11 3047 3053 10.1093/hmg/11.24.3047 12417526

[B23] Atchison FW Capel B Means AR Pin1 regulates the timing of mammalian primordial germ cell proliferation Development 2003 130 3579 3586 10.1242/dev.00584 12810604

[B24] Beck AR Miller IJ Anderson P Streuli M RNA-binding protein TIAR is essential for primordial germ cell development Proc Natl Acad Sci USA 1998 95 2331 2336 10.1073/pnas.95.5.2331 9482885 PMC19335

[B25] Kim B Kim Y Sakuma R Hui C-C Ruether U Jorgensen JS Primordial germ cell proliferation is impaired in Fused Toes mutant embryos Dev Biol 2011 349 417 426 10.1016/j.ydbio.2010.10.010 20969841

[B26] Tanaka SS Toyooka Y Akasu R Katoh-Fukui Y Nakahara Y Suzuki R Yokoyama M Noce T The mouse homolog of Drosophila Vasa is required for the development of male germ cells Genes Dev 2000 14 841 853 10766740 PMC316497

[B27] McLaren A Germline and soma: Interactions during early mouse development Sem Dev Biol 1994 5 43 49 10.1006/sedb.1994.1006

[B28] Armstrong JF Pritchard-Jones K Bickmore WA Hastie ND Bard JB The expression of the Wilms' tumour gene, WT1, in the developing mammalian embryo Mech Dev 1993 40 85 97 10.1016/0925-4773(93)90090-K 8382938

[B29] Pelletier J Schalling M Buckler AJ Rogers A Haber DA Housman D Expression of the Wilms' tumor gene WT1 in the murine urogenital system Genes Dev 1991 5 1345 1356 10.1101/gad.5.8.1345 1651275

[B30] Rackley RR Flenniken AM Kuriyan NP Kessler PM Stoler MH Williams BR Expression of the Wilms' tumor suppressor gene WT1 during mouse embryogenesis Cell Growth Differ 1993 4 1023 1031 8117616

[B31] Englert C Maheswaran S Garvin AJ Kreidberg J Haber DA Induction of p21 by the Wilms' tumor suppressor gene WT1 Cancer Res 1997 57 1429 1434 9108440

[B32] Loeb DM Korz D Katsnelson M Burwell EA Friedman AD Sukumar S Cyclin E is a target of WT1 transcriptional repression J Biol Chem 2002 277 19627 19632 10.1074/jbc.M201336200 11919196

[B33] Kreidberg JA Sariola H Loring JM Maeda M Pelletier J Housman D Jaenisch R WT-1 is required for early kidney development Cell 1993 74 679 691 10.1016/0092-8674(93)90515-R 8395349

[B34] Gao F Maiti S Sun G Ordonez NG Udtha M Deng JM Behringer RR Huff V The Wt1+/R394W mouse displays glomerulosclerosis and early-onset renal failure characteristic of human Denys-Drash syndrome Mol Cell Biol 2004 24 9899 9910 10.1128/MCB.24.22.9899-9910.2004 15509792 PMC525476

[B35] Godin I Wylie C Heasman J Genital ridges exert long-range effects on mouse primordial germ cell numbers and direction of migration in culture Development 1990 108 357 363 2351075 10.1242/dev.108.2.357

[B36] Koff A Giordano A Desai D Yamashita K Harper JW Elledge S Nishimoto T Morgan DO Franza BR Roberts JM Formation and activation of a cyclin E-cdk2 complex during the G1 phase of the human cell cycle Science 1992 257 1689 1694 10.1126/science.1388288 1388288

[B37] Hossain A Saunders GF Role of Wilms tumor 1 (WT1) in the transcriptional regulation of the Mullerian-inhibiting substance promoter Biol Reprod 2003 69 1808 1814 10.1095/biolreprod.103.015826 12855602

[B38] Chen SR Chen M Wang XN Zhang J Wen Q Ji SY Zheng QS Gao F Liu YX The Wilms tumor gene, Wt1, maintains testicular cord integrity by regulating the expression of Col4a1 and Col4a2 Biol Reprod 2013 in press 10.1095/biolreprod.112.105379 23325811

[B39] Runyan C Schaible K Molyneaux K Wang Z Levin L Wylie C Steel factor controls midline cell death of primordial germ cells and is essential for their normal proliferation and migration Development 2006 133 4861 4869 10.1242/dev.02688 17107997

[B40] Ross A Munger S Capel B Bmp7 regulates germ cell proliferation in mouse fetal gonads Sex Dev 2007 1 127 137 10.1159/000100034 18391523

[B41] Richards AJ Enders GC Resnick JL Activin and TGFbeta limit murine primordial germ cell proliferation Dev Biol 1999 207 470 475 10.1006/dbio.1998.9174 10068477

[B42] Chuva de Sousa Lopes SM van den Driesche S Carvalho RL Larsson J Eggen B Surani MA Mummery CL Altered primordial germ cell migration in the absence of transforming growth factor beta signaling via ALK5 Dev Biol 2005 284 194 203 10.1016/j.ydbio.2005.05.019 15993878

[B43] Hartwig S Ho J Pandey P Macisaac K Taglienti M Xiang M Alterovitz G Ramoni M Fraenkel E Kreidberg JA Genomic characterization of Wilms' tumor suppressor 1 targets in nephron progenitor cells during kidney development Development 2010 137 1189 1203 10.1242/dev.045732 20215353 PMC2835332

[B44] Zamboni L Upadhyay S Germ-cell differentiation in mouse adrenal-glands J Exp Zool 1983 228 173 193 10.1002/jez.1402280204 6663256

[B45] Pesce M Farrace MG Piacentini M Dolci S Defelici M Stem-cell factor and leukemia inhibitory factor promote primordial germ-cell survival by suppressing programmed cell-death (apoptosis) Development 1993 118 1089 1094 7505738 10.1242/dev.118.4.1089

[B46] Stallock J Molyneaux K Schaible K Knudson CM Wylie C The pro-apoptotic gene Bax is required for the death of ectopic primordial germ cells during their migration in the mouse embryo Development 2003 130 6589 6597 10.1242/dev.00898 14660547

[B47] De Felici M Scaldaferri ML Lobascio M Iona S Nazzicone V Klinger FG Farini D Experimental approaches to the study of primordial germ cell lineage and proliferation Hum Reprod Update 2004 10 197 206 10.1093/humupd/dmh020 15140867

[B48] Mintz B Russell ES Gene-induced embryological modifications of primordial germ cells in the mouse J Exp Zool 1957 134 207 237 10.1002/jez.1401340202 13428952

[B49] Tam PP Snow MH Proliferation and migration of primordial germ cells during compensatory growth in mouse embryos J Embryol Exp Morphol 1981 64 133 147 7310300

[B50] Koopman P Gubbay J Vivian N Goodfellow P Lovell-Badge R Male development of chromosomally female mice transgenic for Sry Nature 1991 351 117 121 10.1038/351117a0 2030730

[B51] Schmahl J Eicher EM Washburn LL Capel B Sry induces cell proliferation in the mouse gonad Development 2000 127 65 73 10654601 10.1242/dev.127.1.65

[B52] Barrionuevo F Georg I Scherthan H Lecureuil C Guillou F Wegner M Scherer G Testis cord differentiation after the sex determination stage is independent of Sox9 but fails in the combined absence of Sox9 and Sox8 Dev Biol 2009 327 301 312 10.1016/j.ydbio.2008.12.011 19124014

