# Supplementary figures and images for: Disruption of genital ridge development causes aberrant primordial germ cell proliferation but does not affect their directional migration
Source: BMC Biol. 2013 Mar 5;11:22. doi: 10.1186/1741-7007-11-22 (PMC3652777; doi:10.1186/1741-7007-11-22)

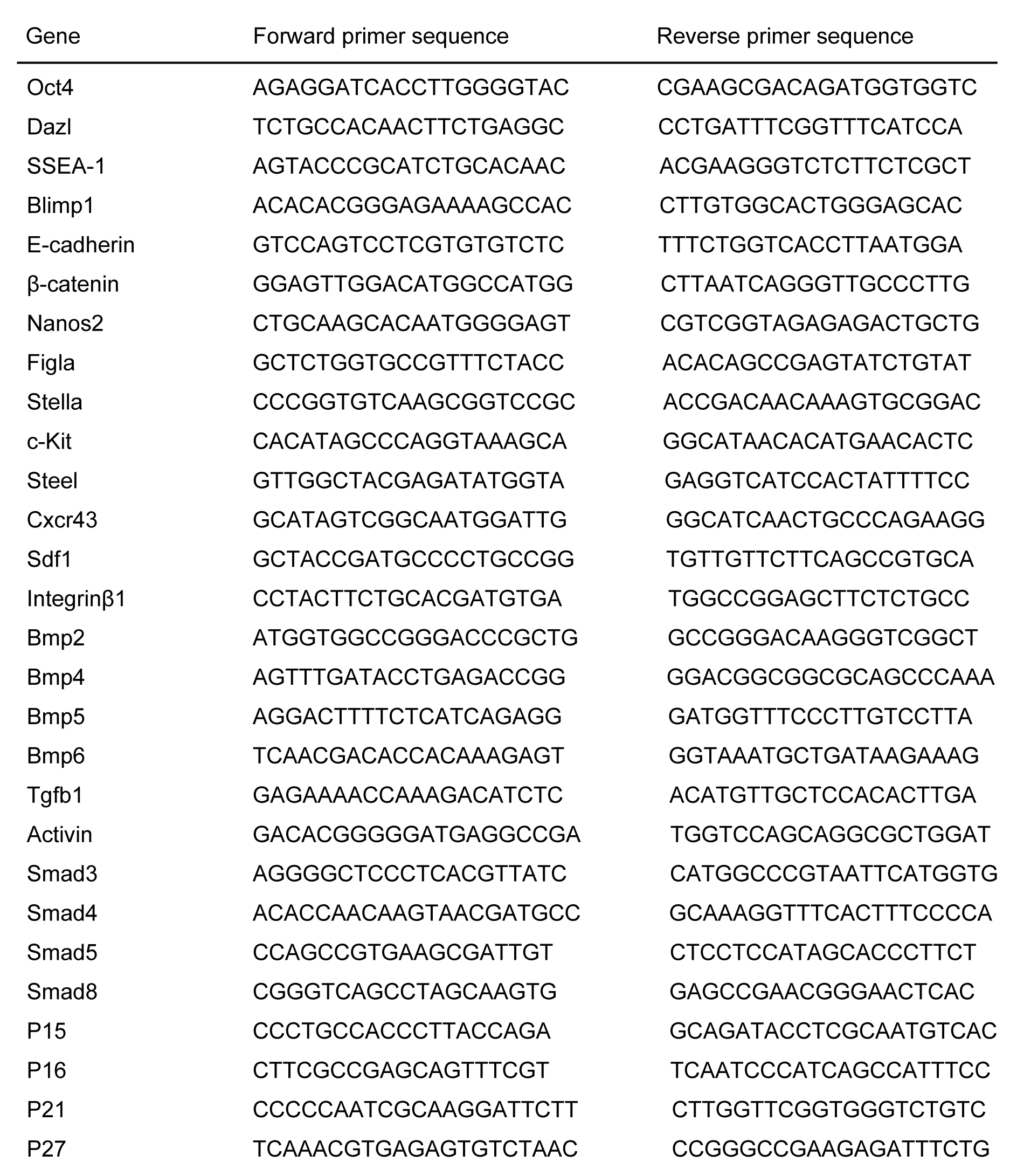

Supplement: Additional file 1 — Table S1: DNA primers used in this study. Primers used in this study for Real-time RT-PCR. [file 1741-7007-11-22-S1.TIFF]
